# Vitamin D Supplementation and Change in Objectively Measured Physical Performance

**DOI:** 10.1093/geroni/igaa057.2739

**Published:** 2020-12-16

**Authors:** Jack Guralnik, Jennifer Schrack, Amal Wanigatunga, Alice Sternberg, Christine Mitchell, Lawrence Appel

**Affiliations:** 1 University of Maryland School of Medicine, Baltimore, Maryland, United States; 2 Johns Hopkins Bloomberg School of Public Health, Baltimore, Maryland, United States; 3 Johns Hopkins School of Public Health, Baltimore, Maryland, United States; 4 Johns Hopkins University School of Medicine, Baltimore, Maryland, United States

## Abstract

Change in usual gait speed (GS) was a prespecified secondary outcome in STURDY and additional physical performance measures were also assessed. Over 12 months, gait speed declined slightly in both the 200IU/d (control) and the intervention groups receiving 1000 or more IU/d, with identical declines in both groups. However, at month 24 GS declined 0.09 m/s from baseline in controls and 0.03 m/s in the intervention group (p=0.03). Subgroup analyses of persons receiving just 1000IU/d throughout the trial compared to controls did not reach significance (p=0.07). Neither SPPB, balance, chair stand tests, or 6-minute walk test showed differences between controls and intervention at any follow-up point. The timed up and go (TUG) test showed a significantly greater improvement in the intervention group at 2 months (p=0.004) but not 12 or 24 months (p=0.17 and 0.79, respectively). Effects of long-term vitamin D supplementation on physical functioning remain unclear.

